# Implementation of the 2018 ESC/ESH Guidelines for the management of hypertension in primary care: the HYPEDIA study

**DOI:** 10.1038/s41371-022-00713-w

**Published:** 2022-07-14

**Authors:** Anastasios Kollias, Emmanouil Foukarakis, Konstantinos Karakousis, The HYPEDIA Study Group, Eleftherios Adamopoulos, Georgios Afaras, Giorgos Aggelopoulos, Theodoros Alexandropoulos, Stavros Alexiadis, Apostolos Alexoudis, Evangelia Anastasiou, Antonios Antoniadis, Ilias Antoniou, Georgios Apazidis, Dimitrios Apostolidis, Georgios Arvanitakis, Panagiotis Arvanitis, Ieronymos Chager, Emmanouil Chalkiadakis, Symeon Charalampous, Christos Charmpas, Thekla Chatziadamidou, Dimitrios Chountis, Ioannis Choursalas, Dimitrios Chrysis, Nikolaos Chrysomallis, Vasiliki Dalakidou, Ioannis Dermitzakis, Ioannis Diakomichalis, Konstantinos Dimitriadis, Nikolaos Dimoulis, Paulos Dolapsakis, Theodoros Douvitsas, Papalymperi Elisavet, Athanasios Efstathiou, Petros Exarchos, Paulos Feggos, Theodoros Feloukas, Dimitrios Floros, Eleni Fourla, Charalampos Fragkiadakis, Marianna Gavriilidou, Dimitrios Georgakopoulos, Euaggelos Georgiadis, Ilias Georgiadis, Kosmas Georgopoulos, Chrysoula Georgopoulou, Emmanouil Giannadakis, Ioannis Giannadakis, Ιgnatios Giavazis, Alexandros Gkalapis, Thomas Gkinis, Dimitrios Eliopoulos, Imprahim Imamoglou, Vaia Ioannidou, Olympia Kapagiannidou, Charalampos Kapernopoulos, Konstantinos Kapetanios, Theodoros Karachalios, Soultana Karakatsani, Eustratios Karanikolas, Dimitrios Karlis, Theodoros Karonis, Andreas Karydakis, Emmanouil Kasotakis, Triantafyllos Katsoulas, Nikolaos Kipouridis, Petros Keryttopoulos, Vasileios Kleiousis, Ioannis Kokkalas, Spyros Kokkinos, Ilias Konstantinidis, Stauros Konstantinidis, Georgios Kontoroupis, Eleni Kosmaoglou, Leonidas Kostalas, Tsampikos Kourtis, Konstantinos Koutrolos, Charikleia Krontira, Kimonas Kypriotakis, Anastasios Kyventidis, Spyridon Lappos, Ioannis Leontaridis, Christos Liavas, Stauros Malliaros, Ioanna Markaki, Georgia Markopoulou-Drosou, Ioannis Mavrepis, Vasilis Mauridis, Fotis Maziotis, Elias Mazokopakis, Alkiviadis Melidoniotis, Nikolaos Maramveliotakis, Anastasia Mitakidou, Dimitrios Mitropoulos, Antonia Moschou, Kyriaki Mousoutzani, Antonis Mperoukas, Kosmas Botsas, Euaggelos Mpougiatiotis, Nikos Mpourneles, Georgios Migias, Savvas Nikiforos, Vasileios Nikolaidis, Christos Nikopoulos, Kadiani Nioti, Nikolaos Oikonomidis, Euaggelos Palmos, Christos Panagos, Maria Pantelidi, Georgios Papadimitriou, Achilleas Papadopoulos, Panagiotis Papadopoulos, Nikolaos Papaioannou, Soultana Papanastasiou, Marianthi Papapavlou, Panagiotis Papas, Vasileios Paulidis, Georgios Pechlivanidis, Ilias Pelekanos, Leonidas Peltekis, Anna Pergaminou, Vasilis Plastiras, Athanasios Platis, Nikolaos Poulopoulos, Petros Prokopis, Ali Risggits, Euaggelos Rosmarakis, Konstantinos Roumpanis, Ioannis Roussis, Alexis Samentzas, Katerina Santipantaki, Periklis Sarafianos, Isidoros Sarris, Dimitrios Savvalas, Georgios Sdralias, Ioannis Sfiniadakis, Simos Siachos, Loukas Sinos, Ourania Sitta, Andreas Skanavis, Ioannis Skias, Panagiotis Skiathitis, Dimitrios Skoutas, Dimitrios Srateh, Ioannis Stathis, Christos Stathopoulos, Christos Staurotheodoros, Emmanouil Stefanakis, Prokopis Stroumpoulis, Konstantinos Svolis, Petros Tapinis, Efstathios Taxiarchou, Maria Thoma, Konstantinos Thomaidis, Michail Timosidis, Paraschos Toloudis, Nikolaos Touroukis, Ioannis Triantafyllidis, Simon Tsalkitzis, Nikolaos Tsamouras, Konstantinos Tsavdaris, Ilias Tserkis, Ioannis Tsiantis, Emmanouil Tsirekas, Ploutarchos Tzavaras, Eutychios Tzemanakis, Aristeidis Tziovas, Aikaterini Vagena, Ioannis Vakalis, Konstantinos Vardakis, Panagiotis Vavoulis, Stefanos Vlachos, Euaggelos Voliotis, Europia Voukelatou, Konstantinos Vrogkistinos, Michail Xafenias, Dionysios Xenos, Ioannis Zacharakis, Natasa Zacharia, Christos Zafeiris, Ioannis Zafeiris, Charilaos Zakopoulos, Vaia Zoi, Ioannis Zolof, George S. Stergiou

**Affiliations:** 1 Hypertension Center STRIDE-7, National and Kapodistrian University of Athens, School of Medicine, Third Department of Medicine, Sotiria Hospital, Athens, Greece; 2 Department of Cardiology, Venizeleio General Hospital, Heraklion, Crete, Greece; 3 Department of Internal Medicine, Larissa General Hospital, Larissa, Greece; 4 Private office, Pieria, Greece; 5 Private office, Athens, Greece; 6 Private office, Aigio, Greece; 7 Private office, Thessaloniki, Greece; 8 Private office, Serres, Greece; 9 Private office, Orestiada, Greece; 10 Private office, Volos, Greece; 11 Private office, Evia, Greece; 12 Private office, Kavala, Greece; 13 Private office, Imathia, Greece; 14 Private office, Arkadia, Greece; 15 Private office, Heraklion, Greece; 16 Private office, Lesvos, Greece; 17 Private office, Karditsa, Greece; 18 Private office, Korinthos, Greece; 19 Private office, Ioannina, Greece; 20 Private office, Kerkira, Greece; 21 Private office, Larissa, Greece; 22 Private office, Alexandroupolis, Greece; 23 Private office, Trikala, Greece; 24 Private office, Komotini, Greece; 25 Private office, Kalamata, Greece; 26 Private office, Patra, Greece; 27 Private office, Lamia, Greece; 28 Private office, Ptolemaida, Greece; 29 Private office, Kastoria, Greece; 30 Private office, Nafplio, Greece; 31 Private office, Kozani, Greece; 32 Private office, Florina, Greece; 33 Private office, Rodos, Greece; 34 Private office, Agrinio, Greece; 35 Private office, Zakynthos, Greece; 36 Private office, Chalkidiki, Greece; 37 Private office, Crete, Greece; 38 Private office, Kilkis, Greece; 39 Private office, Pyrgos, Greece; 40 Private office, Drama, Greece; 41 Private office, Elefsina, Greece; 42 Private office, Sparti, Greece; 43 Private office, Livadeia, Greece; 44 Private office, Xanthi, Greece; 45 Private office, Nafpaktos, Greece; 46 Private office, Chalkida, Greece; 47 Private office, Aridaia, Greece; 48 Private office, Korinthias, Greece

**Keywords:** Hypertension, Risk factors

## Abstract

The HYPEDIA study aimed at evaluating the implementation of the 2018 European guidelines for treating hypertension in primary care. A nationwide prospective non-interventional cross-sectional study was performed in consecutive untreated or treated hypertensives recruited mainly in primary care in Greece. Participants’ characteristics, office blood pressure (BP) (triplicate automated measurements, Microlife BPA3 PC) and treatment changes were recorded on a cloud platform. A total of 3,122 patients (mean age 64 ± 12.5 [SD] years, 52% males) were assessed by 181 doctors and 3 hospital centers. In 772 untreated hypertensives (25%), drug treatment was initiated in the majority, with monotherapy in 53.4%, two-drug combination in 36.3%, and three drugs in 10.3%. Angiotensin receptor blocker (ARB) monotherapy was initiated in 30%, ARB/calcium channel blocker (CCB) 20%, ARB/thiazide 8%, angiotensin converting enzyme inhibitor (ACEi)-based 19%. Of the combinations used, 97% were in single-pill. Among 977 treated hypertensives aged <65 years, 79% had BP ≥ 130/80 mmHg (systolic and/or diastolic), whereas among 1,373 aged ≥65 years, 66% had BP ≥ 140/80 mmHg. ARBs were used in 69% of treated hypertensives, CCBs 47%, ACEis 19%, diuretics 39%, beta-blockers 19%. Treatment modification was decided in 53% of treated hypertensives aged <65 years with BP ≥ 130/80 mmHg and in 62% of those ≥65 years with BP ≥ 140/80 mmHg. Renin-angiotensin system blocker-based therapy constitutes the basis of antihypertensive drug treatment in most patients in primary care, with wide use of single-pill combinations. In almost half of treated uncontrolled hypertensives, treatment was not intensified, suggesting suboptimal implementation of the guidelines and possible physician inertia.

## Introduction

Arterial hypertension is a major global public health problem due to its high prevalence in the general population and its association with considerable cardiovascular morbidity and mortality [1]. The benefits of blood pressure (BP) lowering treatment for prevention of cardiovascular disease are well established. Results of meta-analyses which used data from randomized controlled trials including several hundred thousand patients have shown that a 10/5 mmHg reduction in systolic/diastolic BP is associated with significant reductions in all major cardiovascular events by about 20% [1, 2].

Despite the proven benefit of the antihypertensive treatment, hypertension awareness and BP control rates have remained poor worldwide [1, 3–6]. The 2018 European Society of Cardiology (ESC)/European Society of Hypertension (ESH) guidelines for the management of arterial hypertension identified that poor adherence to treatment and physician clinical inertia (ie, lack of therapeutic action when the patient’s BP is uncontrolled) are important causes of poor BP control [1]. In this context, the 2018 ESC/ESH guidelines adopted an aggressive stepwise treatment strategy using two-drug single-pill combination at the initial step of the treatment algorithm for most patients [1]. Moreover, angiotensin-converting enzyme inhibitors (ACEi) and angiotensin receptor blockers (ARB) constitute the basis of the combination therapy due to their established cardiovascular and renal protective effects together with their excellent tolerability [1]. The initiation of treatment in most patients with a two-drug single-pill combination improves adherence as well as speed, efficiency, and predictability of BP control, resulting in improvement of the hypertension control rates in the general population [7].

Office BP measurement has been regarded as the “cornerstone” for diagnosis and management of hypertension, because the vast majority of the evidence on the risks associated with elevated BP and the benefits of treatment-induced BP lowering has been based on office BP measurements [1, 8, 9]. Despite the limitations of office BP and the increasing use of out‐of‐office BP using home and less so ambulatory monitoring, at present and for some time to come it is likely that in many people the diagnosis and management of hypertension will be based on office BP measurement alone, especially in primary care [8, 9]. The 2018 ESC/ESH guidelines propose office BP threshold levels for the antihypertensive drug treatment initiation and for treatment targets [1]. Moreover, stricter BP treatment target ranges are recommended for patients aged <65 years (120–129/70–79 mmHg, systolic/diastolic) [1].

As hypertension affects more than one third of the adult population, most of these patients are managed in primary care. Real-life data on hypertension control rates in primary care setting demonstrate an evidence practice gap which is multifactorial and mainly attributed to poor patients’ compliance, physician inertia, and issues within the healthcare system services such as patient access and treatment costs [1, 5, 6]. The purpose of the present study was to assess the therapeutic approach in newly diagnosed untreated and previously treated hypertensive patients by primary care doctors in Greece, with regard to the 2018 European guidelines.

## Materials/Subjects and methods

This was a national level, non-interventional, observational, cross-sectional, epidemiological study conducted in a single visit for each participant. The study was carried out between September 2019 and April 2020.

This study was designed to assess doctor’s practices in the management of hypertension and to determine whether these practices adhere to the 2018 ESC/ESH recommendations.

Fully ambulatory patients aged ≥18 years, diagnosed with essential hypertension at the time of enrolment were eligible for inclusion. Primary care sites and hospital centers were selected on the basis of distribution criteria so as to cover several territories throughout the country, and aiming to include hypertensive patients from different geographical regions of Greece. All the candidate sites were invited and informed by phone calls and/or emails. Each primary care investigator and each hospital clinic was asked to recruit at least 15 sequential patients of whom 25% naïve to treatment and 75% previously treated.

Medical history, drug treatment, BP measurements and intended changes were recorded for each participant in a single visit using an electronic patient’s form of an online cloud platform. This electronic form was identified by a code assigned exclusively to each participant and was filled in during the study visit. Triplicate office BP measurements were taken after 5 min sitting rest at a chair with back support at 1-minute intervals, with the arm resting on a table (mid-arm at heart level) and feet flat on the floor. BP measurements were performed using a validated automated oscillometric, upper-arm BP monitor (Microlife BPA3 PC, Widnau, Switzerland), equipped with a medium-large size cuff (wide range 22–42 cm) [10]. BP readings were automatically transferred to personal computer (wired transfer) and synchronously uploaded to the online cloud system with the patient’s electronic form simultaneously opened so as to allow matching of data. In accordance with the 2018 ESC/ESH guidelines the average of the second and third BP measurement was used [1]. Uncontrolled hypertension in treated patients was defined as office BP ≥ 130/80 mmHg (systolic and/or diastolic) in those aged <65 years and ≥140/80 mmHg in ≥65 years [1]. All medical examinations recorded in this observational study were carried out as recommended for routine clinical practice.

The study protocol was approved by the Scientific/Ethics Committee of the Sotiria Hospital (Athens, Greece), General Hospital of Larissa (Greece), and Venizeleio General Hospital of Crete (Greece). All participants signed written informed consent for their participation in the study.

The Kolmogorov–Smirnov test was used to check the normal distribution of the study variables. Continuous variables were expressed as mean (SD). Comparison of qualitative variables between 2 groups (i.e., between the hospital hypertension centers and the primary sector) was performed using Chi-squared or Fisher’s exact test. Comparison of quantitative variables between 2 groups was performed using the t-test or Mann-Whitney test based on normal or non-normal distribution respectively. Binary logistic regression analysis was applied to identify determinants of uncontrolled hypertension among treated patients. Independent variables included the setting of care (primary care or hospitals), and factors related to patients’ characteristics (age, sex, body mass index [BMI], diabetes mellitus, smoking status, educational status). Non-parametric values were natural log-transformed before being used in the above analysis. Statistical analysis was performed using the IBM SPSS Statistics (Version 21.0. Armonk, NY IBM Corp). A p-value lower than 0.05 was considered statistically significant.

## Results

Overall, a total of 3122 patients were enrolled, of whom 3002 (25% untreated) were recruited by 181 primary care doctors and 120 (21% untreated, *p* = NS vs primary care) in 3 hospital centers. The study participants’ characteristics are shown in Table 1.

**Table 1:** Demographic and clinical characteristics of study participants (mean ± SD or %).

Variable	
Age (years)	64.0 ± 12.5
Males	52.4%
Body mass index (kg/m^2^)	28.4 ± 4.6
Diabetes mellitus	24.7%
Smoking	26.6%
Coronary heart disease	12.8%
Treated hypertension	75.3%
Number of antihypertensive drugs	2.0 ± 0.9
Office systolic blood pressure (mmHg)	142.5 ± 19.3
Office diastolic blood pressure (mmHg)	83.8 ± 12.1
Heart rate (bpm)	74.6 ± 12.6

**Fig. 1:**
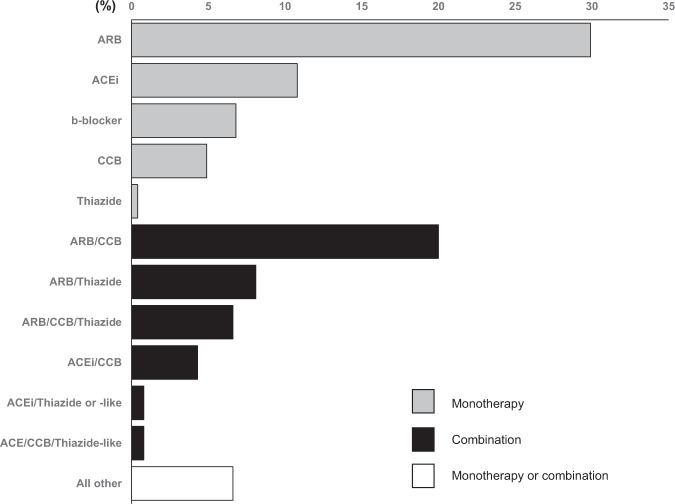
Choices regarding drug treatment initiation in untreated hypertensive patients. ARB angiotensin receptor blockers, ACEi angiotensin-converting enzyme inhibitors, CCB calcium channel blockers.

In 772 untreated hypertensives (25% of the overall population; mean age [SD]: 58 [12] years), mean systolic/diastolic BP was 149.7 ± 18.1/89.0 ± 11.6 mmHg. Drug treatment was initiated in 769 patients with drug monotherapy in 53.4%, two-drug combination in 36.3%, and three drugs in 10.3%. There were 3 primary care doctors who initiated triple-drug therapy in 80–100% of their patients. Main choices for treatment initiation included angiotensin receptor blocker (ARB) monotherapy in 30%, ARB/calcium channel blocker (CCB) 20%, ARB/thiazide 8%, angiotensin-converting enzyme inhibitor (ACEi) monotherapy 11%, ACEi/CCB 4%, ACEi/thiazide (or thiazide-like) 1%, CCB monotherapy 5% and beta-blocker monotherapy 7% of the cases (Fig. 1). Of the combinations used, 97% were prescribed as a single-pill combination.

In 329 untreated patients aged <80 years with systolic BP ≥ 150 mmHg, treatment was initiated with drug monotherapy in 32%, two-drug combination in 50%, and three drugs in 18%.

In 2350 treated hypertensives (75% of the overall population), mean systolic/diastolic BP was 140.2 ± 19.1/82.1 ± 11.8 mmHg. Mean duration of hypertension since initial diagnosis was 8.6 ± 6.9 years and treatment included on average 2.0 ± 0.9 antihypertensive drugs. Main drug classes administered in these patients were ARB monotherapy 18%, ARB/CCB 15%, ARB/thiazide 12%, ARB/CCB/thiazide 12%, ACEi monotherapy 7%, ACEi/CCB 4%, ACEi/thiazide (or thiazide-like) 3%, ACEi/CCB/thiazide-like 2%, CCB monotherapy 5%, beta-blocker monotherapy 4%, and other choices 18%. Single pill combinations were used in 85% of patients receiving combination therapy.

**Fig. 2:**
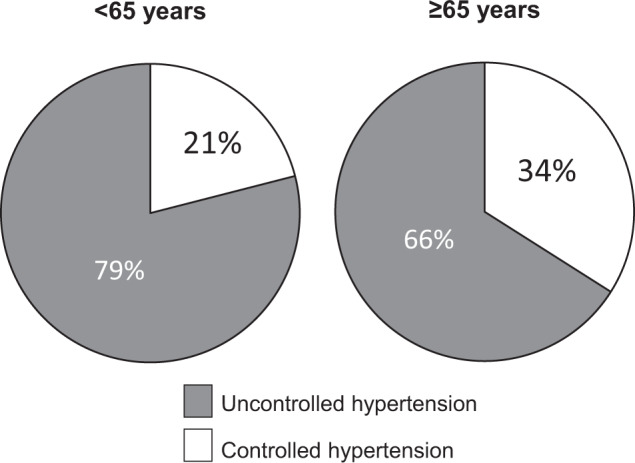
Blood pressure control rates. Percentage of uncontrolled and controlled hypertension among 2350 treated hypertensive patients according to office blood pressure targets recommended by 2018 European guidelines (<130/80 mmHg in <65 years; <140/80 mmHg in ≥65 years).

Among treated hypertensives aged <65 years (*N* = 977), 79% had BP ≥ 130/80 mmHg (systolic and/or diastolic), whereas among those aged ≥65 years (*N* = 1373) 66% had BP ≥ 140/80 mmHg (Fig. 2). Treatment modification (dose increase, drug addition, use of fixed combinations, switch of drug to another within the same or other class) was decided in 53% of the treated hypertensives aged <65 years with BP ≥ 130/80 mmHg, and in 62% of those ≥65 years with BP ≥ 140/80 mmHg. There were no significant differences in the types of treatment decisions per care of setting (primary care versus hospital centers).

Binary logistic regression for identifying determinants of uncontrolled BP (≥130/80 mmHg in treated patients aged <65 years and ≥140/80 mmHg in those ≥65 years), showed that natural logarithm of BMI (OR 10.9, 95% CI 1.9, 62.8), and male versus female sex (OR 2.2, 95% 1.3, 3.8), were associated with a higher probability of uncontrolled hypertension.

For patients aged <65 years, 70% of primary care and 82% of hospital doctors used a systolic BP target <130 mmHg (*p* = 0.06 for difference), whereas for diastolic BP target <80 mmHg 58% and 55% respectively (*p* = NS). For patients aged ≥65 years, 98% of primary care and 100% of hospital doctors used a systolic BP target <140 mmHg (*p* = NS), whereas for diastolic BP target <80 mmHg 61% and 51%, respectively (*p* = NS).

## Discussion

This observational study assessed the treatment strategy of the doctors in the routine clinical practice in Greece compared with the 2018 ESC/ESH guidelines for the management of arterial hypertension. The main findings include the following: (i) Renin-angiotensin system blockers constitute the basis of antihypertensive treatment, mainly in single-pill two-drug combinations; (ii) in a considerable proportion of newly diagnosed untreated patients, treatment was initiated with monotherapy; (iii) more than half of treated patients had uncontrolled BP; (iv) treatment modification was decided in only about half of treated patients with uncontrolled BP.

Renin-angiotensin system blockers (ARB, ACEi) are recommended in the first step of the 2018 ESC/ESH treatment algorithm [1]. This is supported by the strong evidence showing cardiovascular and renal protection with these drugs classes [1]. This study showed that in newly diagnosed hypertensives, ARB or ACEi monotherapy, or ARB- or ACEi-based combination therapy represent the most common choices. The same was valid regarding the treatment choices in treated hypertensives. There are additional interesting observations regarding the use of ARBs and ACEis. First, ARBs were much more commonly used than ACEis. This probably reflects the rather comparable outcome efficacy of these drug classes combined with more favorable tolerability profile of ARBs [11]. Second, there was a trend for a more frequent prescription of ARB or ACEi in combination with CCB rather than with a diuretic. This observation could reflect the influence of the findings of the ‘Avoiding Cardiovascular Events through Combination Therapy in Patients Living with Systolic Hypertension’ (ACCOMPLISH) trial in favor of the ACEi combination with CCB versus diuretic [12]. Interestingly, the 2020 guidelines by the International Society of Hypertension support the use of ARB or ACEi with CCB rather than a diuretic [13], yet this publication was too recent to have influenced clinical practice. Third, the ARB- or ACEi-based combinations were mainly used as single-pill combinations. This is particularly important since single-pill combinations improve adherence and persistence in treated hypertensives [14].

Another interesting finding is the relatively frequent use of drug monotherapy for treatment initiation in newly diagnosed hypertensive patients. These data are somewhat in contrast to the 2018 ESC/ESH strategy recommending two-drug combination therapy at first step for most patients aged <80 years with systolic BP ≥ 150 mmHg [1]. These findings might reflect suspected white-coat reaction in some cases and hesitance of the doctors to decide, or clinical inertia. Real life data from a large population-based cohort in Italy showed that a significant number of patients who received initial monotherapy failed to move to combination treatment, as recommended by the 2018 ESC/ESH guidelines, highlighting considerable therapeutic inertia in primary care [5]. This is highly important since randomized-based trial evidence suggests that antihypertensive combination treatment of any complexity (up to three or more drugs) is more protective compared to no-treatment or less-complex treatment [15]. On the other hand, in 10% of patients, their doctors initiated therapy with a 3-drug combination and in fact 3 primary care doctors adopted this strategy in 80–100% of their patients. Although the doses of the drugs were not available and low-dose combinations might have been used, this aggressive strategy is not supported by the 2018 guidelines. While it is expected to lead to faster and better control rates, it might result in overtreatment with excessive BP decline and its associated adverse effects.

A large proportion of treated patients in the current study presented with uncontrolled hypertension based on office BP, thus failing to reach the ESC/ESH recommended BP targets. It is also important to note that demographic characteristics such as male sex and higher BMI, which add cardiovascular risk, were associated with higher probability of uncontrolled hypertension. Although the ESC/ESH guidelines emphasize the important role of out-of-office BP measurements in the management of hypertension, reality is that in many patients in primary care the management of hypertension is based solely on office BP. Moreover, the 2018 ESC/ESH guidelines recommend a stricter BP target goal for patients <65 years (<130/80 mmHg) [1]. In this study, despite the high rate of uncontrolled hypertension, therapeutic decision to modify treatment, was taken in only about half of the uncontrolled patients. In addition, a considerable number of doctors used less strict BP goals compared with that of the 2018 ESC/ESH guidelines. All the above findings indicate suboptimal implementation of the ESC/ESH guidelines which is attributed to several factors, probably related to inadequate patients’ adherence and physicians’ therapeutic inertia in achieving optimal hypertension control.

When interpreting the findings of the current study it should be taken into consideration that BP was assessed in single office visit and out-of-office BP was not evaluated. Yet, in real life general practice, office BP measurements, usually under poorly defined conditions, may constitute the sole BP assessment method for many hypertensives considering the lack of time and resources in primary care. In addition, the doctors might have been influenced by (i) their participation in the study, which may have improved their compliance to the guidelines, and (ii) the fact that they were forced to take triplicate BP measurements (automated measurements with validated device and online storage) which may lead to more standardized office BP assessment compared to the usual care measurements. However, the study findings suggesting physician inertia indicate that the true behavior of physicians in clinical practice has been revealed to a large extent.

In conclusion, renin-angiotensin system blocker-based therapy, mainly ARBs, constitutes the basis of antihypertensive drug treatment in the vast majority of patients in primary care, with single-pill combinations being widely used. In almost half of treated uncontrolled hypertensives treatment was not intensified (physician inertia), suggesting suboptimal implementation of the 2018 ESC/ESH goals in primary care. These findings support the need of reinforcing information and education strategies for primary care doctors so as to perform standardized BP measurements, detect hypertensive patients of high cardiovascular risk and implement early, more intense and simplified therapy through wider use of double and triple fixed-dose combinations, in line with the recommendations. Thus, it is highly important that both doctors and patients are informed about the evidence-based safety and benefits of the antihypertensive treatment strategy according to current guidelines, the need of accurate diagnosis, and the importance of achieving optimal control of BP in terms of more efficient cardiovascular disease prevention.

### Summary Table

#### What is known about the topic

- Previous studies have confirmed poor control rates of treated hypertension in primary care worldwide.
- 2018 ESH/ESC guidelines for the management of hypertension recommended a stepwise treatment algorithm with the use of single-pill combinations and stricter blood pressure targets than previous guidelines.

#### What this study adds

- In a large sample of hypertensive patients in primary care the implementation of the 2018 ESH/ESC guidelines for the management of hypertension was found to be suboptimal.
- Initial drug monotherapy, instead of drug combination, was decided for a considerable proportion of newly treated hypertensives.
- In almost half of treated uncontrolled hypertensives, treatment was not intensified, suggesting physician inertia.

## Data Availability

The study data can be available after reasonable request from the corresponding author and Menarini Hellas S.A.
